# Comparative Evaluation of Mucosal Vibrator with Topical Anesthetic Gel to reduce Pain during Administration of Local Anesthesia in Pediatric Patients: An *in vivo* Study

**DOI:** 10.5005/jp-journals-10005-1523

**Published:** 2018-08-01

**Authors:** Sandeep Tandon, Garima Kalia, Meenakshi Sharma, Rinku Mathur, Khushboo Rathore, Mahima Gandhi

**Affiliations:** 1 Senior Professor and Head, Department of Pedodontics and Preventive Dentistry Government Dental College, Jaipur, Rajasthan, India; 2 Postgraduate Student, Department of Pedodontics and Preventive Dentistry Government Dental College, Jaipur, Rajasthan, India; 3 Assistant Professor, Department of Pedodontics and Preventive Dentistry Government Dental College, Jaipur, Rajasthan, India; 4 Assistant Professor, Department of Pedodontics and Preventive Dentistry Government Dental College, Jaipur, Rajasthan, India; 5 Postgraduate Student, Department of Pedodontics and Preventive Dentistry Government Dental College, Jaipur, Rajasthan, India; 6 Postgraduate Student, Department of Pedodontics and Preventive Dentistry Government Dental College, Jaipur, Rajasthan, India

**Keywords:** Facial pain rating scale, Lignocaine jelly, Pain scores, Sound, eye, motor scale.

## Abstract

**Introduction:**

Usually discomfort and pain are associated with dental work, especially for young patients. Pain control can be achieved by using anesthesia. Sight of injection can terrify any patient and if the patient is a child it is really difficult to convince them for injections. Alternatives to injections have been explored. Pediatric dentists are using anesthesia in the form of jelly and patch. Recently, the concept of mucosal vibration has been put forward to enhance the effectiveness of local anesthesia.

**Aim:**

The aim of the present study was to compare and evaluate the effectiveness of lignocaine jelly and mucosal vibration in reducing pain during administration of local anesthesia in pediatric dental patients.

**Materials and methods:**

Thirty children in the age group 6 to 11 years who required bilateral anesthesia for dental treatment in mandible were selected for this study. Pain was compared using Wilcoxon signed-rank test at the time of injection using Sound, Eye, Motor (SEM) scale as objective criteria and facial pain rating (FPR) scale as subjective criteria after administration of injection by a trained assistant who was blinded to the procedure.

**Results:**

Local anesthetic injection along with mucosal vibration resulted in significantly less pain (p = 0.001) in comparison with the injections without the use of mucosal vibration.

**Conclusion:**

The result shows that mucosal vibration can be used as an effective means to reduce the intensity of pain during local anesthetic injection in dentistry.

**How to cite this article:** Tandon S, Kalia G, Sharma M, Mathur R, Rathore K, Gandhi M. Comparative Evaluation of Mucosal Vibrator with Topical Anesthetic Gel to reduce Pain during Administration of Local Anesthesia in Pediatric Patients: An *in vivo* Study. Int J Clin Pediatr Dent 2018;11(4):261-265.

## INTRODUCTION

Pain is defined as an unpleasant sensory and emotional experience arising from actual or potential tissue damage or described in terms of such damage.^1^ In pediatric dentistry, pain sensation is generated by stimuli like sound of the drill or touch of the needle at the time of local anesthetic administration and is not necessarily dependent on tissue damage.^2^ Injection is the most common cause of anxiety in pediatric patients which affects the quality of dental treatment. Ointments, anesthetic sprays, gels, or adhesive patch are topical application of local anesthetic which are utilized to reduce pain of local anesthetic injections, but these methods have their own limitations.^3^

**Fig. 1:**
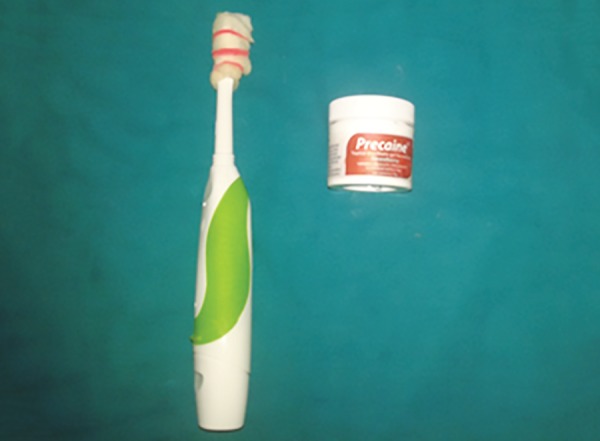
Rajasthan University of Health Science mucosal vibrator

Recently, the concept of vibration stimuli along with local anesthetic injection has been introduced. Mucosal vibration is a nonpharmacological technique used for reducing pain associated with local anesthetic injection. The explanation of analgesic effect of vibration by the gate control theory of pain was given by Melzack and Wall.^4^ They proposed that the pain sensation can be reduced by activating nerve fibers that conduct touch, pressure, and temperature stimuli. The spinal cord is believed to have a neurological “gate” that either blocks or permits pain signals to travel up the spinothalamic tract to the brain. Stimulation of the larger diameter fibers through touch signal mechanoreceptors (e.g., massage techniques, rubbing, pressure, ice packs, acupuncture, or vibration) causes activation of inhibitory neurons, which prevent the activation of projection neurons at the synaptic junction in the dorsal horn of the spinal cord. This results in a closure of the gate causing pain sensation.^5^ A decrease in pain intensity during vibratory stimulation was observed in patients suffering from acute or chronic musculoskel-etal pain of various origins in the study conducted by Lundeberg et al.^5^ Earlier use of Vibraject, attached to a traditional syringe to transfer a vibrating stimulus to the needle, did not result in a significant reduction of pain scores during needle insertion.^6,7^ Recently, Dr Steven Goldberg introduced Dental vibe^®^ (Dental Vibe Inc.), which delivers vibration as a counterstimulation and in a sustained frequency onto the site of injection.^3^ Implementing the same concept of vibration, we have used Colgate 360° Total Advanced sonic-powered toothbrush for mucosal vibration. Bristles of the brush were covered with soft sponge with a frequency of 20,000 strokes/minute vibrations and named it as Rajasthan University of Health Science (RUHS) mucosal vibrator (Fig. 1). Mechanism of action is the same as that of Dental Vibe. Mucosal vibrator decreases patient anxiety in case the patient is afraid of injection. The soft sponge over bristles has a massaging effect which helps in dissolution of solution faster and a soothing effect. The present study was thus conducted to compare the effectiveness of mucosal vibrator with lidocaine topical anesthetic gel during local anesthetic administration.

**Fig. 2:**
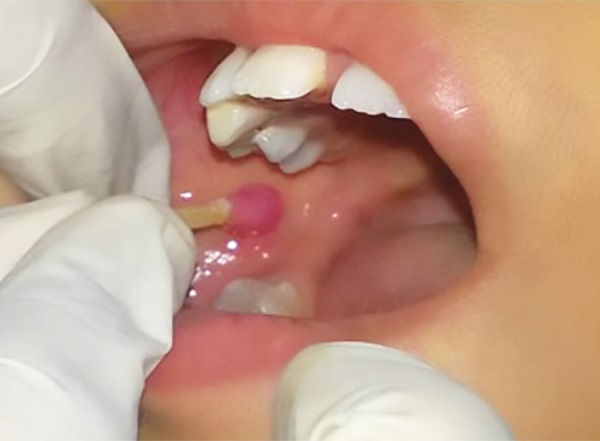
Topical anesthetic application

## MATERIALS AND METHODS

**Fig. 3:**
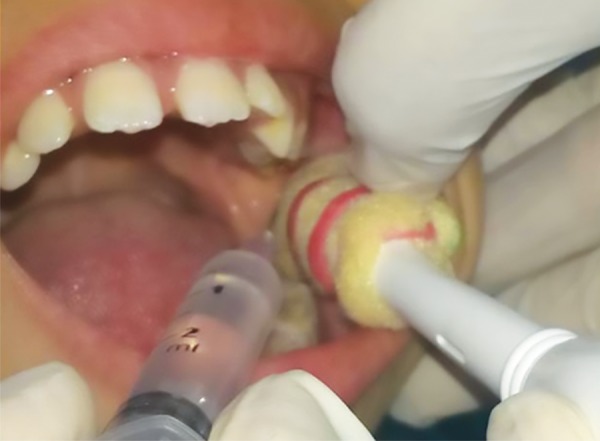
Administration of local anesthetic with RUHS mucosal vibrator

Tand Preventive Dentistry Department in RUHS College of Dental Sciences, Jaipur. Subjects were randomly chosen from the outpatient department. A random crossover design was used. Each child thus served as his/her own control; 30 children were included in each group, group I (with mucosal vibration) and group II (with topical anesthetic). The inclusion criteria were patients with a mean age of 8.5 ± 1.88 (6-11) years with Frankel’s behavior rating scale positive or definitely positive and who required bilateral local anesthetic injections in lower jaw for dental treatment whether restoration, pulpotomy, pulpectomy or extraction. Patients with Frankel’s behavior rating scale as negative or definitely negative and medically compromised patients were excluded from the study. Parents of the patients were explained about the study. An informed consent was obtained. The protocol of the study was reviewed and approved by the Medical Research Ethical Committee of Rajasthan College of Dental Sciences, Jaipur. In the first appointment, the injection site was isolated using cotton roll and the topical Precaine (8% lignocaine and 0.8% dibucaine) (Pascal International™) anesthetic gel was applied using sterile cotton dipped applicator for 30 seconds to the injection site (Fig. 2) and left for 3 to 5 minutes after informing the child, then 1.5 mL of local anesthetic solution was deposited (1 mL/min) using a 24 gauge sterile hypodermic syringe needle and the dental treatment was carried out. In the subsequent appointment after 4 to 5 days on the contralateral side of the same arch, after tell-show-do technique, RUHS mucosal vibrator was applied before and during the local anesthetic administration to the injection site for 1 minute. The local anesthesia was administered by keeping the needle in close vicinity to the vibrator and the vibration continued for 15 seconds after the removal of the needle. It had a massaging effect and helped in the dissipation of the local anesthetic solution (Fig. 3).

**Table 1:** **Table 1:** Sound, eye, motor scale

		*Comfort or pain level*							
*Possible indications of pain*		*1—comfort*		*2—mild discomfort*		*3—moderately painful*		*4—painful*	
Sound		No sounds indicating pain		Nonspecific sounds; possible indication of pain		Specific verbal complaints (such as “ow”), raises voice		Verbal complaint indicates intense pain (such as screaming, sobbing)	
Eye		No eye signs of discomfort		Eyes wide, show of concern, no tears		Watery eyes, eyes flinching		Crying tears running down face	
Motor		Hands relaxed; no apparent body tension		Hands showing some distress or tension; grasping of chair owing to discomfort, muscular tension		Random movement of arms or body without aggressive intention of physical contact, grimacing, twitching		Movement of hands to make aggressive physical contact (such as pushing, pulling head away)	

**Fig. 4:**
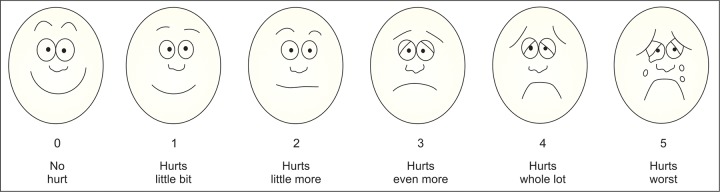
Wong-Baker face scale (faces pain rating scale)

As it is extremely difficult to quantify pain in children, two different objective and subjective scales were used:

1. The SEM scale (Table 1), an objective scale, was used to measure pain. An assistant was trained to measure and calibrate the SEM scale. The assistant was blinded to avoid bias.^8,9^
2. The second scale was Wong-Baker FPR scale (Fig. 4), a subjective scale used to assess pain. A set of six cartoon faces were shown to the child with varying facial expressions ranging from a very smiling face to a very sad face. A brief explanation was given to the child about each face after which the child was instructed to choose the face that best described his/ her feelings while receiving local anesthesia.^10,11^ Video recording of the whole procedure was done for further future evaluation.

### Statistical Analysis

Wilcoxon signed-rank test was used for statistical analysis. All analysis was conducted using Statistical Package for the Social Sciences software (p-value of <0.05 was considered statically significant).

## RESULTS

There was a statistically significant difference between the mucosal vibration and the topical gel group (p < 0.001). Table 2 shows the comparison between the mean of pain intensity with mucosal vibration and gel group according to SEM Scale. Table 3 shows the comparison between the mean of pain intensity with mucosal vibration and gel group according to FPS scale (Fig. 4).

**Table 2:** **Table 2:** Comparison between the pain score with mucosal vibration and with topical anesthesia according to FPS scale using Wilcoxon signed-rank test

*Groups*		*Mean age*		*Mean pain score (mean ± SD)*		*p-value*	
With mucosal vibration (group I) n = 30		8.5 ± 1.88		2.1 ± 0.78		0.001***	
With topical anesthetic (group II) n = 30		8.5 ± 1.88		4.7 ± 0.87			

SD: Standard deviation; ***Statistically significant

## DISCUSSION

Management of pain during dental treatment is the most critical subject. Pain sensation is initiated by condition stimuli as sound of the drill or touch of the needle during local anesthetic injections and is not necessarily dependent on tissue damage.^2^ The pain due to injection of local anesthetic can be decreased by a number of methods which include reducing the speed of injection, application of counterirritation, varying the rates of infiltration, distraction techniques, buffering and warming the local anesthesia, use of fine needles with improved syringes, precooling the injection site, application of topical analgesics, and use of vibration.^7^ The present study was conducted to compare and evaluate the efficacy of mucosal vibration with topical local anesthetic Precaine gel (Pascal International™) in pain reduction during injection of local anesthesia. A random crossover design was planned. In the study, patients with Frankel’s behavior rating scale as positive and definitely positive were included to rule out the emotional factors, such as tension, anxiety, depression, worries, and previous eventful dental experiences from affecting the results of study, as these may open the pain gate, thus reducing the threshold of pain.^11,12^

**Table 3:** **Table 3:** Comparison between the pain score with mucosal vibration and with topical anesthesia according to SME scale using Wilcoxon signed-rank test

*Groups*		*Mean age*		*Mean pain score (mean ± SD)*		*p-value*	
With mucosal vibration (group I) n = 30		8.5 ± 1.88		1.4 ± 0.68		0.001***	
With topical anesthetic (group II) n = 30		8.5 ± 1.88		3.2 ± 0.79			

SD: Standard deviation; ***Statistically significant

In this study, topical anesthetic gel was selected for comparison with the mucosal vibration because it is frequently used to reduce pain during an injection pro-cedure.^7^ Minasian and Yagiela^13^ in a study concluded that topical anesthesia is more effective if charged ions of an anesthetic agent were driven by tissue through iontophoresis before insertion of needle.

Toxic sequel may be associated with topical anesthetic because some of the topical agents are relatively toxic and amount of drug absorbed through the mucosa. Furthermore, the taste of the sprays and gels can make the child uncooperative. The effectiveness of the topical anesthetic gel also gets reduced due to dissolution of the agents with the saliva at the site. This was supported in a study conducted by Shilpapriya et al.^7^ Also, the additional time required to apply topical anesthesia may increase the child’s apprehension concerning the approaching procedure.^14^ Due to such problems, a predictable means of pain control along with local anesthetic administration is desirable.

Kincheloe et al^15^ studied 77 dental patients and found that a topical anesthetic (unspecified), when applied for 3 minutes, was no more efficient in reducing the pain of injection than was placebo.

The gate control theory of pain given by Melzack and Wall^4^ serves as a basis for explaining the analgesic effect of mucosal vibration. According to the theory, A-β nerve fibers, which transmit signal from vibration and touch receptors in the skin, stimulate inhibitory interneurons of the spinal cord. These neurons decrease the pain signal transmitted by A-δ and C fibers from the skin to second-order neurons that ascend to the brain.^16^

Studies conducted by Shilpapriya et al,^7^ Nasehi et al,^16^ and DiFelice et al^17^ have reported decrease in pain when vibrating stimuli were applied during local anesthetic injection.

No statistically significant difference was found in the study conducted by Saijo et al^18^ in pain perception when comparison was done between VibraJect^®^ and a conventional syringe along with anaject.

The present study showed that the use of mucosal vibrator during local anesthetic injection reduces patient discomfort and the soft sponge covering of the bristles of tooth brush used as mucosal vibrator in the study can have a massaging effect which helps in the dissolution of solution faster and a soothing effect.

Mucosal vibration can be an effective alternative for reduction in pain due to injection. The vibration might also be more effective if a more efficient vibration device compared with foam swab was employed. The level of dental anxiety may have a strong influence on an individual’s reaction, and knowledge of each patient’s level of dental anxiety may help tailor treatment to the patient’s needs.^19^

The present study included limited number of patients. Similar study involving larger sample size is required. Future studies can be conducted to compare the effectiveness of RUHS mucosal vibrator with similar devices to reduce pain perception during local anesthetic injection.

## CONCLUSION

Mucosal vibrator is an effective device during local anesthetic injection administration to alleviate pain and stress of injection. This device contributes both physiological and psychological aspect of patient management based on gate control theory of pain, audible distraction, and massaging effect of the device. As very few studies have been conducted related to this and since the sample size taken in this study was small, therefore, further research is warranted with a large sample size.
